# Organotypic Modeling of the Tumor Landscape

**DOI:** 10.3389/fcell.2020.606039

**Published:** 2020-11-24

**Authors:** Maria M. Haykal, Clara Nahmias, Christine Varon, Océane C. B. Martin

**Affiliations:** ^1^Université Paris-Saclay, Institut Gustave Roussy, Inserm U981, Biomarqueurs Prédictifs et Nouvelles Stratégies Thérapeutiques en Oncologie, Villejuif, France; ^2^Univ. Bordeaux, INSERM, BaRITOn, U1053, Bordeaux, France

**Keywords:** tumor microenvironment, cancer, 3D model, therapies, metastasis, tumor dissemination

## Abstract

Cancer is a complex disease and it is now clear that not only epithelial tumor cells play a role in carcinogenesis. The tumor microenvironment is composed of non-stromal cells, including endothelial cells, adipocytes, immune and nerve cells, and a stromal compartment composed of extracellular matrix, cancer-associated fibroblasts and mesenchymal cells. Tumorigenesis is a dynamic process with constant interactions occurring between the tumor cells and their surroundings. Even though all connections have not yet been discovered, it is now known that crosstalk between actors of the microenvironment drives cancer progression. Taking into account this complexity, it is important to develop relevant models to study carcinogenesis. Conventional 2D culture models fail to represent the entire tumor microenvironment properly and the use of animal models should be decreased with respect to the 3Rs rule. To this aim, *in vitro* organotypic models have been significantly developed these past few years. These models have different levels of complexity and allow the study of tumor cells alone or in interaction with the microenvironment actors during the multiple stages of carcinogenesis. This review depicts recent insights into organotypic modeling of the tumor and its microenvironment all throughout cancer progression. It offers an overview of the crosstalk between epithelial cancer cells and their microenvironment during the different phases of carcinogenesis, from the early cell autonomous events to the late metastatic stages. The advantages of 3D over classical 2D or *in vivo* models are presented as well as the most promising organotypic models. A particular focus is made on organotypic models used for studying cancer progression, from the less complex spheroids to the more sophisticated body-on-a-chip. Last but not least, we address the potential benefits of these models in personalized medicine which is undoubtedly a domain paving the path to new hopes in terms of cancer care and cure.

## Introduction

Carcinogenesis is a complex multistep process, often described as somatic evolution. Typically, cancer progression involves the accumulation of genetic and/or epigenetic somatic modifications and exposition to environmental factors. Indeed, the development of many tumors is tightly linked with genotoxicity, chronic infections, dietary habits, or autoimmunity; which are all underlined by inflammation. Early on, Fearon and Vogelstein (1990) described a sequence of defined genetic events driving the formation of colorectal cancers. Afterward, the seminal works of Hanahan and Weinberg (2000, 2011) contributed to shift cancer research from a reductionist point of view with a sole focus on the cancer cell itself to a more comprehensive view involving cues from the neighboring niche. Therefore, carcinogenesis is the fruit of the interplay between multiple cell autonomous and non-autonomous processes, defined as “Hallmarks of cancer,” that include genomic instability, proliferative abnormality, stromal reprogramming, angiogenesis, immune suppression and tumor sustaining inflammation. In the following sections, we first define the tumor microenvironment (TME) and briefly depict its different components. We also summarize the recently described interactions between the TME actors and the tumor cells in the cancer progression cascade. In depth understanding of such interactions renders necessary the study of tumor cells within their microenvironment, as this is crucial for cancer progression. In this line of thought, we describe the most promising organotypic models used for modeling cancer progression stages from the initial tumor and its microenvironment to dissemination and metastasis.

## Part I—Role of the Microenvironment in Tumoral Progression

The importance of the tumor microenvironment is embodied in the concept that cancer cells do not cause the disease alone, but rather corrupt recruited and neighboring normal cell types to serve as accessories to the crime (Hanahan and Coussens, 2012). In particular, interactions between cancer cells and their microenvironment represent a powerful relationship that influences disease initiation and progression and patient prognosis. For decades, the focus of cancer research has been almost exclusively on epithelial tumor cells. However, in the past few years, there has been a major shift toward the study of the TME, elucidating that tumor progression is dependent on an intricate network of interactions among cancer cells and their surroundings (McAllister and Weinberg, 2014; Taniguchi and Karin, 2018; Hinshaw and Shevde, 2019).

Tumors are unquestionably heterogenous entities, composed of phenotypically distinct cellular populations with different functions. This is illustrated by the clonal evolution theory (Nowell, 1976), TME heterogeneity (Junttila and de Sauvage, 2013) and hierarchal organization of cancer cell subpopulations that includes cancer stem cells (CSCs) and their progenies. Some studies have shown that CSCs are the driving force of tumor formation as they exhibit self-renewal and tumor−initiating capacities and phenotypic plasticity. Plasticity offers cancer cells the ability to switch from a differentiated state to an undifferentiated CSC-like state, responsible for long term tumor growth and drug resistance. Recently, observations of anatomically distinct niches of CSCs within tumors have emerged (reviewed in Plaks et al., 2015; Batlle and Clevers, 2017). These niches could have a role in preserving the plastic phenotype of CSCs and their protection from the immune system. Nonetheless, the heterogeneous tumor is a part of a larger society comprising many other actors that define the tumor microenvironment.

### Defining the Tumor Microenvironment

The tumor microenvironment, a diversified compartment of differentiated and progenitor cells, comprises all the non-malignant host cellular and non-cellular components of the tumor niche including, but not restricted to, endothelial cells, adipocytes, cells of the immune and nervous systems, and the stroma.

#### Non-stromal Components

##### Endothelial Cells

The most well-known extrinsic modulator of cancer cell growth is neovascularization (Folkman, 1985). Early studies using mouse models show that the angiogenic switch increases the proliferation rate of cancer cells (Folkman et al., 1989). Angiogenesis is crucial to the ability of tumors to thrive and the vascular endothelium is an active participant in the formation of a growth-permissive tumor microenvironment. Vascularization is driven by the hypoxic center of the tumor where hyperproliferation results in increased oxygen demand. Consequently, low oxygen induces the expression of angiogenic proteins like vascular endothelial growth factor (VEGF) and basic fibroblast growth factor (bFGF) (Papetti and Herman, 2002) that activate endothelial cells and attract them toward the tumor to form new vessels, allowing the delivery of nutrients and oxygen. Without angiogenesis, tumors are condemned to quiescence and cell death. Tumor vascularization requires the cooperation of different TME cells, mainly vascular endothelial cells that provide structural integrity to the newly formed vessels and pericytes that ensure their coverage and maturity (Weis and Cheresh, 2011). Endothelial cells also constitute routes to metastatic dissemination via angiogenesis and contribute to resistance to chemotherapies through an overexpression of drug efflux pumps thereby decreasing the tumor’s access to the drug (Hida et al., 2013).

##### Adipocytes

Cancer-associated adipocytes (CAAs) support cancer growth mainly through secretion of adipokines like adipsin (Goto et al., 2019), chemerin (Lu et al., 2019) as well as proinflammatory cytokines (Dirat et al., 2011) and growth factors. CAAs also supply lipids for cancer cell membranes and organelles, induce metabolic reprogramming in cancer cells and provide proteases for cancer cell invasion (reviewed in Deng et al., 2016). Moreover, through the production of tumor-promoting cytokines and factors, they have been shown to confer resistance to hormone therapies, chemotherapies, radiotherapies and targeted therapies in breast cancer (Choi et al., 2018), and to contribute to tumor progression across a variety of obesity-associated cancers (Park et al., 2014) such as esophagus, gastric, liver, kidney, colorectal, pancreatic, breast, ovarian, prostate, and thyroid cancers. Adipocytes from white adipose tissue are recruited to tumors, can differentiate into pericytes and incorporate into vessel walls contributing to angiogenesis and to tumor proliferation (Zhang et al., 2012).

##### Infiltrating Immune Cells

Variations in immune profiles are linked to prognosis and therapeutic responses (Gentles et al., 2015). All adult solid tumors contain infiltrates of diverse immune cell subsets that influence pro-tumorigenic and antitumor phenotypes. Of all infiltrating myeloid immune subsets, tumor-associated macrophages (TAMs) best represent this paradigm. TAMs are abundant in all stages of tumor progression and can be polarized into inflammatory M1 or immuno-suppressive M2 macrophages, depending on microenvironment stimuli (Ruffell and Coussens Lisa, 2015). While a subset of TAMs has antitumoral effects, others stimulate cancer cell proliferation by secreting growth factors, produce proteolytic enzymes that digest the ECM to facilitate tumor cell dissemination, and provide a supportive niche for metastatic tumor cells (Mantovani and Allavena, 2015). Eosinophils, primitive actors of innate immunity, have been shown to infiltrate tumors and influence tumor progression. Activated eosinophils secrete IL-10 and IL-12, to inhibit cancer cells growth, or can mediate cell death by direct cytotoxicity (Gatault et al., 2015; Lucarini et al., 2017). However, they can also promote tumor growth by secreting growth factors such as epidermal growth factor (EGF) and transforming growth factor-β1 (TGF-β1) (Grisaru-Tal et al., 2020). As tumors grow, myeloid-derived suppressor cells (MDSCs) (Kumar et al., 2016), immunosuppressive precursors of macrophages and dendritic cells (DCs), promote tumor vascularization and disrupt major mechanisms of immunosurveillance, including tumoral antigen presentation, T cell activation and cytotoxicity (Lindau et al., 2013).

The other major subset of tumor infiltrating immune cells is of lymphoid origin and includes T lymphocytes and natural killer (NK) cells. T lymphocytes can be grouped into 3 major subtypes: (i) T_H_ lymphocytes divided mainly in two lineages: pro-inflammatory T_H__1_ and anti-inflammatory T_H__2_; (ii) Regulatory T cells (T_reg_), primarily pro-tumorigenic *via* their immunosuppressive activity; and (iii) cytotoxic T cells (T_C_) that destroy tumor cells through granzyme and perforin mediated apoptosis (Fridman et al., 2012; Lindau et al., 2013). A third lineage of effector T_H_ cells, characterized by IL-17 secretion, called T_H__17_ cells, acts as double-edged sword in anti-tumor immunity and tumorigenesis (Alizadeh et al., 2013).

##### Nerve Cells

Peripheral nerves are a common feature of the TME and emerging regulators of cancer progression. Innervated tumors are aggressive, have high proliferative indices and an increased risk of recurrence and metastasis (Magnon et al., 2015). Cancer cells can grow around nerves and invade them in a process called perineural invasion, which represents yet another route for dissemination (reviewed in Jobling et al., 2015). Recently, Zahalka et al. (2017) have shown that adrenergic nerves promote angiogenesis by activating the angiogenic switch in endothelial cells. Moreover, many studies described the formation of new nerve endings within tumors, showing that they stimulate their own innervation, a process termed axonogenesis, by expressing neurotrophic factors (Wang et al., 2014; Huang et al., 2015) or releasing exosomes containing axonal guidance molecules (Madeo et al., 2018). In return, nerves provide the tumor with neurotransmitters that enhance cancer cell growth.

#### Stromal Components

In healthy tissues, the stroma constitutes the main barrier against tumorigenesis. However, transformed cancer cells can direct stromal reprogramming to support tumor growth and progression.

The stroma is composed of the extracellular matrix (ECM) and specialized connective tissue cells, including fibroblasts, and mesenchymal stem cells.

##### The Extracellular Matrix

The ECM constitutes the scaffold of tissues and organs, providing the essential signals to maintain tissue architecture and to regulate cell growth and apoptosis. It is a complex network of glycoproteins, proteoglycans, glycosaminoglycans and other macromolecules. About 300 different proteins have been classified as ECM proteins, in what is called the matrisome (Hynes and Naba, 2012). The ECM undergoes constant remodeling by different actors, mainly enzymes such as collagenases and matrix metalloproteases (MMPs) and by fibroblasts. ECM stiffening, induced by increased collagen deposition and crosslinking, disrupts tissue morphogenesis contributing to malignant progression, but also facilitates metastasis and infiltration of immune cells in tumor sites (Bonnans et al., 2014).

##### Cancer-Associated Fibroblasts

Fibroblasts are widely distributed in all tissues. They constitute a multifunctional cell type residing in the ECM, shaping it by secreting collagens and fibrous macromolecules but also degrading it by releasing proteolytic enzymes, like MMPs.

Fibroblasts are known to modulate immune response by recruiting leucocyte infiltration and regulating inflammation *via* the secretion of growth factors, cytokines and chemokines and to play an important role in maintaining tissue homeostasis (Buckley et al., 2001). During wound healing or fibrosis, another type of specialized fibroblasts called myofibroblasts is present in the tissue (Tomasek et al., 2002). Tumors, for long considered as wounds that do not heal, are associated with a stroma similar to that observed in wound healing called the activated stroma, where fibroblasts resemble myofibroblasts and are called cancer-associated fibroblasts (CAFs). The activated stroma supports cancer progression (Hanahan and Coussens, 2012). Importantly, as for cancer cells, it has been described that CAF population is highly heterogeneous with tumor-promoting or tumor-suppressing CAFs and personalized anticancer therapies targeting CAFs could be of great interest (reviewed in Liu et al., 2019; Mhaidly and Mechta-Grigoriou, 2020).

##### Mesenchymal Stem Cells

The definition and characteristics of mesenchymal stem cells (MSCs) have been a matter of debate for a long time, and their characterization is an active field of research (Nombela-Arrieta et al., 2011). It is now established that MSCs are multipotent progenitor cells originating from the bone marrow that can migrate systemically through blood vessels and differentiate into osteoblasts, chondrocytes, or adipocytes. To date, the primary functions of MSCs within the TME are to regulate the immune response by the release of immunomodulatory cytokines and to promote tissue regeneration. Owing to their multipotent and cell fusion properties, they can also be at the origin of vascular cells, contributing to angiogenesis, of myofibroblasts and more rarely of cancer cells themselves.

### Crosstalk Between Tumor Cells and Components of the TME in Cancer Progression

**FIGURE 1:**
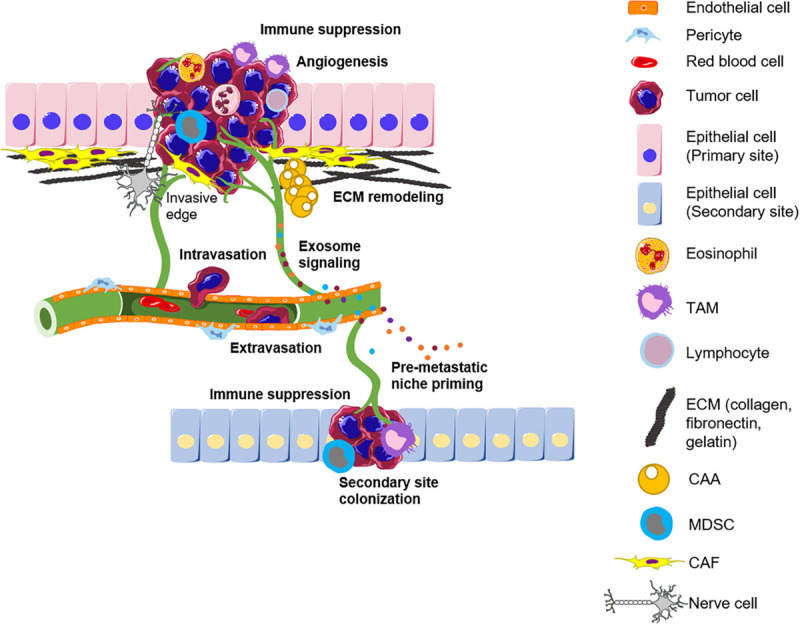
The tumor microenvironment influences the different stages of cancer progression. The primary tumor is infiltrated by different immune subsets and surrounded by a remodeled matrix. Angiogenesis ensures tumor growth by supplying nutrients and also provides a route for metastasis. Intravasation of tumor cells into blood vessels allows their shuttling to a novel site. The secondary site is primed by exosomes secreted by tumor cells and the different actors of the TME to allow the successful seeding of incoming tumor cells. TAM, Tumor-associated macrophage; ECM, Extracellular matrix; CAA, Cancer-associated adipocytes; MDSC, Myeloid-derived suppressor cells; CAF, Cancer-associated fibroblast; TME, Tumor microenvironment.

The tumor microenvironment plays a critical role in determining tumor fate, and stromal reprogramming has been recognized to be critical for carcinogenesis (Mantovani et al., 2008). Rudolf Virchow first proposed the possibility of a link between chronic inflammation and tumorigenesis in the nineteenth century after the observation of infiltrating leukocytes within tumors. This is now considered a hallmark of cancer. Cancer progression is associated with an ever-evolving tissue interface of direct epithelial–stromal interactions that regulate cancer cell metastasis and disease progression. This section describes the complex crosstalk between the actors of the TME and the cancer cells that take place during the different stages of cancer progression from the early cell autonomous events to the late metastatic stages (Figure 1).

#### Primary Tumor Progression

Cancer cells reprogram the tumor-infiltrating stromal and immune cells, which facilitates primary tumor growth and progression. Therefore, it is important to decipher the reciprocal crosstalk between cancer cells and their heterotypic microenvironment.

Epithelial cancer progression is influenced by the cells’ contact with immune cells and a carcinogen-exposed stroma (Barcellos-Hoff and Ravani, 2000), by an overexpression of metalloproteases (Fukuda et al., 2011) which create a suitable environment for invasion, or by the stimulation with altered stromal cells like CAFs. In the skin, epigenetic modifications of fibroblasts are induced by ultraviolet exposure, leading to the production of inflammatory cytokines and matrix-remodeling enzymes that together influence the formation of epithelial tumors (Hu et al., 2012). CAFs accumulate in the TME along with tumor growth (Kalluri, 2016) and are activated by cytokines and growth factors of the TME, such as TGF-β (Taniguchi et al., 2020) and Fibroblast Growth Factor (FGF). In their turn, CAFs provide growth factors like VEGF to enhance angiogenesis and vascular permeability (Fukumura et al., 1998). Furthermore, TAMs can support many aspects of tumoral progression. They can secrete mediators that enhance tumor cell survival and proliferation such as growth factors and cytokines [epidermal growth factor (EGF), interleukin 6 (IL-6), and tumor necrosis factor (TNF) (Noy and Pollard Jeffrey, 2014)].

Another crucial step for cancer progression is immune evasion. This is supported mainly by the action of MDSCs. These cells infiltrate the developing tumors and inhibit the mechanisms of immune editing of cytotoxic immune cells, all the while promoting tumor vascularization (Talmadge and Gabrilovich, 2013). TAMs can also promote cancer immune escape by displaying immunosuppressive functions (Noy and Pollard Jeffrey, 2014). Other myeloid cells including neutrophils, monocytes, and eosinophils infiltrate the tumor and promote tumor growth by inhibiting antitumor immunity. Neutrophils can even induce genotoxic damages (Wilson et al., 2015) or recruit tumor-promoting T_H__17_ lymphocytes (Ortiz et al., 2015). Additionally, invasion of the basement membrane underlying the epithelium by the tumor cells is a basic step for the upcoming dissemination. For this, CAFs have a physical impact on tumors that results in increased ECM stiffness around tumor cells and consequent mechanical stress. TAMs are also capable of driving invasive phenotypes (Condeelis and Pollard, 2006). In breast cancer, they facilitate invasion of tumor cells by sustaining a signaling paracrine loop involving CSF-1 and EGF (Goswami et al., 2005), and by the secretion of proteases (Gocheva et al., 2010). Thus, once the tumor cells evade the host immune system and gain the ability to invade the surrounding tissue, metastatic dissemination of cancer cells can take place.

#### Metastatic Dissemination

Metastasis is the leading cause of mortality among cancer patients (Mehlen and Puisieux, 2006). In epithelial tumors, metastasis begins with the cellular invasion of the basement membrane and the subsequent migration of cancer cells into the blood stream. One of the initial steps for primary tumor invasion is epithelial-mesenchymal transition (EMT). Under the influence of various signals, mainly TGF-β, cells gradually lose their epithelial traits while gaining mesenchymal ones that confer migratory capacities (Mani et al., 2008). CAFs participate in a TGF-β and platelet-derived growth factor (PDGF) signaling crosstalk with tumor cells to support EMT and the acquisition of an invasive phenotype (van Zijl et al., 2009). EMT can also enable the acquisition of CSC traits (Mani et al., 2008), suggesting that not only it causes cancer cells to disseminate from the primary tumor but also can provide these cells with the self-renewal properties needed for their subsequent implantation at secondary sites. Although CSCs are not be the only cells responsible for metastasis, the CSC-generated hierarchy of stem-like and differentiated tumor cells is able to initiate metastatic growth (Merlos-Suárez et al., 2011). However, EMT is not the only mechanism used by epithelial cells for migration. Epithelial cancer cells can migrate as single cells, as loosely attached cords or as highly organized collective entities (reviewed in Friedl et al., 2012). During early stages of cancer migration, CAFs increase the production of collagen in the underlying stroma and the fibers become aligned, giving rise to a stiffer ECM hence allowing the migration of cancer cells away from the primary tumor (Conklin et al., 2011). This is largely mediated by CAFs secreted factors that stiffen the ECM, namely enzymes of the Lysyl Oxidase (LOX) family (Kalluri and Zeisberg, 2006).

During metastasis, cancer cells cross the endothelial barrier during a step called **intravasation** to enter the blood stream, and by **extravasation** to exit from circulation into distant tissues, processes that involve different receptors, a plethora of signaling pathways, and interactions with the actors of the surrounding microenvironment (Reymond et al., 2013). Intravasation seems to require the cooperative work of a triad consisting of macrophages that localize to blood vessels where they help tumor cells intravasate into the blood stream (Harney et al., 2015). However, despite the help of macrophages, only 0.01% of cells that intravasate form detectable metastases (Chambers et al., 2002). Cancer cells in the blood stream can be shielded by platelets from NK-mediated cytotoxicity (Palumbo et al., 2005), and platelet binding enhances cancer cell adhesion to vessel wall and subsequent extravasation (Zhang et al., 2011; Schumacher et al., 2013). Inflammation also modulates endothelial crossings through TNF-induced vascular permeabilization, cyclooxygenase 2 (COX2)-dependent prostaglandin production and MMP-mediated tissue remodeling.

#### Secondary Organ Colonization

Docking of cells in organs to form secondary tumors is not a random process. Organ tropism has been first described by Stephen Paget in 1886 as the “seed-and-soil” theory, in which he suggests that metastasis is not the fruit of hazard but tumors have clear organ preferences for secondary colonization. Paget’s theory gave the basis for the description of the premetastatic niche: the primary tumor executes preparative events, preceding detectable metastasis, that render the secondary milieu less hostile for colonization by cancer cells. Studies of the premetastatic niche are still in their infancy but some traits and events are now clearer. Settlement of tumor cells at distant sites is dependent on tumor-secreted cytokines and extracellular vesicles, like exosomes, that enable the premetastatic microenvironment to support their colonization (Liu and Cao, 2016). These tumor-secreted factors communicate to both hematopoietic and mesenchymal stem cell compartments. It has been shown that bone marrow-derived VEGFR1+ cells are already present in premetastatic sites before tumor cell arrival, suggesting the communication between primary and secondary sites (Kaplan et al., 2005). Seeding is also facilitated by the LOX-mediated fibronectin upregulation in resident fibroblasts and recruitment of myeloid cells (Erler et al., 2009). Neutrophils may also be involved in the priming of metastatic sites. Neutrophils accumulate in premetastatic livers of mice bearing colorectal tumors (Wang et al., 2017) and their accumulation has been shown to be required for pancreatic cancer metastasis (Steele et al., 2016). Recently, it was also shown that omentum resident macrophages are required for ovarian cancer metastasis (Etzerodt et al., 2020). Neutrophils also serve as an energy source to fuel metastatic tumor cells. In a breast cancer model, infiltrating neutrophils are induced to store lipids upon interaction with resident mesenchymal cells in the lung so that when disseminated tumor cells (DTCs) arrive, neutrophils transfer their stored lipids to DTCs for their survival and proliferation (Li et al., 2020).

Colonization of secondary tissues requires the same elements as growth of the primary tumor namely, sufficient nutrients and oxygenation. One important step for metastatic tumor cell survival is the reversal to an epithelial phenotype *via* mesenchymal–epithelial transition (MET) to regain the ability of proliferation and differentiation. Once tumor cells colonize the secondary site, genetic instability inherent in neoplastic cells continues to operate at each cell division, and these cells continue the remodeling of the site, just as described above.

Accordingly, the crosstalk between cancer cells and their microenvironment provides valuable insights into cancer formation, progression and spread. Hence, it is necessary to study cancer as a whole process by modeling the interactions between tumor cells and their microenvironment to improve development of new therapies against cancer progression and metastasis.

## Part II—Organotypic *in vitro* Models

### Advantages of 3D Models Over 2D Models and Animal Experiments

Cancer research has long been based on two-dimensional (2D) cell culture, mainly in order to earn the right of passage to *in vivo* experiments. Conventional 2D cell cultures allowed the study of many mechanisms that drive tumor growth and the evaluation of optimal drug doses and toxicities. However, currently available cell lines fail to represent the genetic background across the range of human cancers (Huang A. et al., 2020) and may adapt to growth in culture, rather than mimic the behavior of the tumor in a complex microenvironment. Because they also lack all elements of the tumor stroma and surrounding tissue, they fail to mimic the complexity of the tumor microenvironment (Gillet et al., 2011). Owing to this, a large gap exists between the knowledge obtained in these models compared to *in vivo* cancer models because results of 2D experiments rarely predict therapeutic response in animals. This can be explained by the fact that cells cultured in 2D do not have the same architecture as cells *in vivo* that are arranged in three-dimensional (3D) structures unattached to planar surfaces. Furthermore, cultured monolayers lack the capacity to mimic *in vivo* tumoral hypoxia and exhibit a very different metabolism. Consequently, cells in monolayer cultures proliferate at unnaturally rapid rates (Langhans, 2018), differ in gene/protein expression compared to *in vivo* models, and alter their dynamic processes such as cell division and migration (Duval et al., 2017).

Even though *in vivo* experiments have the advantage of being physiologically relevant in contrast to cells cultured out of their bodily context, they have many flaws (Day et al., 2015). Aside from being long, expensive and ethically questionable, the use of human cancer cells in mouse models mostly requires the use of immunocompromised mice that lack, to varying extents, the immune components, thus limiting the advantages of these approaches in modeling tumoral progression and response to drugs. Indeed, the inflammatory immune cell component is lacking in immunocompromised mice. Although the engrafted tumors may exhibit a stromal response with the growth of endothelial cells and fibroblasts, these stromal cells originate from mice and therefore the implication of human TME could not be extrapolated. Moreover, it has recently been shown that patient-derived xenografts (PDXs) present genomic instability with continuously changing copy number alterations landscapes, and so their passaging causes a drift from the original tumor (Ben-David et al., 2017). As such, mouse co-clinical trials using PDXs have shown very little progress beyond proof of concept due to logistical issues (Clohessy and Pandolfi, 2015).

Even with strong supporting preclinical evidence, many targeted therapies produce modest clinical results, a fact now highlighted by the tremendous National Lung Matrix Trial that assessed personalized medicine in non–small cell lung cancer (NSCLC) (Middleton et al., 2020). The results have been fairly disappointing with a response rate of only 10% with some abandons due to lack of treatment efficacy. Genetically engineered mouse models of NSCLC, used for preclinical studies, have mutational burdens more than 100-fold lower than that of human disease (McFadden et al., 2016) arguing for the use of more appropriate preclinical models that integrate the immune and stromal landscapes beyond the genetic aberrations.

Another issue resides in the ability to translate results of immunotherapy from bench to clinic because of the high failure rate observed in human clinical trials after promising results obtained in mouse models. Even the durable clinical benefits observed with immune checkpoint blockers (ICBs) in some tumor types have been seen in a minority of patients (Cardin et al., 2014; Herbst et al., 2014; Hammel et al., 2016). Given the complexity of the tumor microenvironment, it is imperative to create models that include different immune cell types the administered compound may interact with.

Efforts have been made these last few years to “humanize” the mouse’s immune system by grafting human hematopoietic stem cells in mice or by transgenic expression of Human Leucocyte Antigen (HLA) (reviewed in Shultz et al., 2012; De La Rochere et al., 2018). However, the high cost of recipient mice, scarcity of human bone marrow acquisitions, engraftment variability, and laborious technical demands represent high inconveniences in a preclinical setting. Hence, optimal mouse studies are very cumbersome for simultaneous evaluation of numerous drugs and may be inefficient due to the different metabolic processing of drugs between humans and mice. Thus, high-throughput *in vitro* screening systems are essential precursors to *in vivo* evaluations. Developing 3D organotypic models that recapitulate physiological functions would allow further replacement and reduction of animal models as recommended by the 3Rs rule^1^.

*In vitro* 3D cultures recapitulate much better the architecture of tissues and capture the complexity of solid tumors than 2D counterparts, all the while allowing the modeling of different stages of the carcinogenic process (Yamada and Cukierman, 2007; Tanner and Gottesman, 2015). The concentric arrangement of cells in 3D cultures resembles initial avascular stages of solid tumors *in vivo* and non-vascularized micro-metastatic foci. More sophisticated 3D cultures also include different elements of the TME; allowing their use to study cellular interactions within tumors and to model stages of cancer progression. Additionally, genome-wide screens performed on 3D cultures showed improved detection of cancer genes and pathways compared with those performed in 2D (Han et al., 2020). Thus, increased biologically relevant behavior and characteristics could be acquired from genetic editing in organoids, cocultures, and 3D growth models. Moreover, the coalition between biologists, bioengineers and physicians inspired many strategies to reproduce *ex vivo* the complexity of biological systems. These approaches mimic organ topography, mechanical forces of tumor cells, matrix stiffness, functionality, and complexity much better than 2D or even 3D culture systems (van Duinen et al., 2015).

**FIGURE 2:**
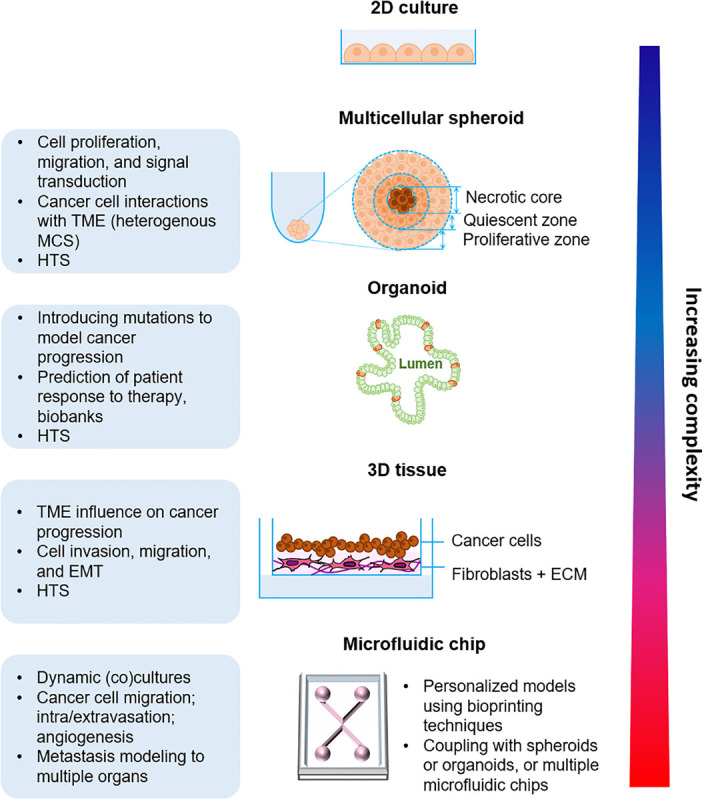
Organotypic *in vitro* models of cancer progression with increasing biological complexity from the simple multicellular spheroid model to the more complex microfluidic approaches. TME, Tumor microenvironment; MCS, Multicellular spheroid; HTS, High-throughput screening; ECM, Extracellular matrix.

In the following section, we will describe the existing *in vitro* organotypic models for cell culture (Figure 2).

### Overview of *in vitro* Organotypic Cellular Models

#### Multicellular Spheroids

Multicellular spheroids (MCSs) or 3D cellular aggregates represent the bridge that fills the gap between 2D cultures and more elaborate 3D techniques. They are fairly representative of the *in vivo* situations because of their heterogeneity as they are composed of proliferating, non-proliferating, well-oxygenated, hypoxic and necrotic cells. Other features of MCSs like cell-cell signaling and interactions, the presence of different cellular layers, the genetic expression profiles, and drug resistance patterns are similar to characteristics of the natural cellular conditions. Currently, there exists many techniques for MCS production such as the forced floating methods in non-adherent plates, hanging drop method, the use of scaffolds and matrices, or even more sophisticated methods using microfluidic systems (reviewed in Ferreira et al., 2018).

MCS can be used for tumoral modeling by either forming homogenous cultures using solely cancer cells, or by more sophisticated cultures using cancer cells with components of the TME like fibroblasts, endothelial cells (Andrique et al., 2019) or immune cells, hence forming heterotypic spheroids. Encapsulating MCS in biomimetic hydrogel scaffolds offers biophysical and biochemical cues that simulate the behavior of extracellular matrix, essential for regulating cancer cell behavior (Li and Kumacheva, 2018).

#### Organoids

The term organoid, meaning resembling an organ, was first used in 1946 by Smith and Cochrane to describe a case of cystic teratoma. Ever since, it has been inaccurately used to describe some cell structures and aggregates, but the actual definition is now clearer: an organoid is a collection of organ-specific cell types that develops from stem cells, that possesses a minima of specific organ functions, and self-organizes to mimic the architecture of the organ itself (reviewed in Lancaster and Knoblich, 2014). Early pioneering works of Mina Bissell showed that primary epithelial cells derived from mouse mammary glands could self-organize into glandular structures and secrete milk proteins (Lee et al., 1984). These advances were followed by the works of Clevers’ lab, that described the generation of intestinal crypt organoids from Lgr5+ stem cells (Sato et al., 2009).

Now, it is recognized that organoids can be generated using two types of stem cells: **pluripotent stem cells (PSCs)** which can be embryonic or induced pluripotent stem cells or **adult stem cells (ASCs)** that reside in adult tissues and are tissue-specific, cultured under specific growth factor cocktails that allow their long-term expansion by mimicking the organ stem cell niche. To date, organoids have been developed for many organs including intestine (Spence et al., 2011), kidney (Takasato et al., 2015), brain (Lancaster et al., 2017), liver (Camp et al., 2017), stomach (McCracken et al., 2014), pancreas (Hohwieler et al., 2017), ovary (Kessler et al., 2015), and lung (Dye et al., 2015) among others. These organoids have been used for multiple approaches such as high-throughput drug screening efficacy and toxicity, host-microbe interactions and infectious diseases (Bartfeld et al., 2015; Leslie et al., 2015; Garcez et al., 2016), and disease modeling (reviewed in Dutta et al., 2017) in particular tumor development, which will be later discussed in detail.

Epithelial organoids recapitulate many aspects of organ development and disease and represent many opportunities for cancer modeling and anticancer drug testing. However, it is important to note the existence of some drawbacks and limitations. Organoids lack the native organ microenvironment: the stromal compartment, immune cells and vascularization, and they are mostly cultured in poorly defined animal matrices. Although, novel synthetic analogous ECM may constitute a better alternative as they are controllable and permit fine tuning of matrix constituents (Gjorevski et al., 2016).

#### 3D-Tissues

The recreation of simple tissues has been described in a cell sheet engineering method using cells grown to confluence on culture dishes grafted with a temperature-responsive polymer, poly-(N-isopropylacrylamide). This technique allows cell growth at 37°C and cell harvest at room temperature as intact cell sheets and subsequently the stacking of different sheets to generate heterotypic thin 3D tissue analogs. Using this technique vascularized tissues (Asakawa et al., 2010) and liver tissue-like structures (Kim et al., 2012) were obtained.

Organotypic epithelial raft cultures, originally developed to study keratinocytes (Fuchs, 1990), represent an interesting approach to study epithelial cancer cell behavior, notably cancer cell invasion. These cultures are mechanically supported by semipermeable inserts and are either submerged in medium or maintained at an air–liquid interface. Epithelial tissues can be constructed in stages by first embedding stromal cells, mainly fibroblasts, for several days followed by seeding of epithelial cells on top (Kalabis et al., 2012), or also embed immune cells within the layers to obtain an integral tissue (Huang et al., 2017). These cultures generate a stratified tissue resembling the epithelium seen *in vivo* with a proliferating basal layer and differentiating supra-basal layers. The use of 3D-tissue revealed some advantages compared to organoids when the access to the epithelial cells’ apical surfaces is needed, for example to study host-pathogen interactions. To illustrate this using a colonic 3D-tissue, Martin and colleagues have shown that infection with genotoxin-producing *Salmonella* enterica synergises with the loss of APC to promote genomic instability and carcinogenesis (Martin et al., 2019). Although it should be noted that an elegant recent study has described the possibility to revert organoid polarity allowing access to the apical surfaces of the cells (Co et al., 2019). Miniaturized 3D tissues can be used to facilitate high-throughput drug screening (Dutta et al., 2017).

#### Microfluidic Approaches

The static nature of nutrients and metabolites in 3D cultures isn’t representative of the physiological conditions due to the lack of fluid shear stress and hydrostatic pressure that can greatly influence cell behavior (Polacheck et al., 2011). Microfluidic systems, based on the progress in synthetic biology, have enabled the development of *in vitro* assays that facilitate the study of cellular behavior under a spatiotemporally controlled microenvironment in which molecular, biophysical and cellular components can be tuned according to physiologically relevant parameters. These microfluidic cell culture systems, known under the term organ-on-a-chip, are usually made of continuously perfused hollow microchannels populated by living cells (reviewed in Bhatia and Ingber, 2014). To date, many organs have been successfully modeled in microfluidic devices. One of the first models was lung alveoli that responded to bacterial infection and inflammation (Huh et al., 2010), but also that reflected drug toxicity (Huh et al., 2012). Many studies followed that assessed nephrotoxicity in human kidney tubules-on-a-chip (Jang et al., 2013), liver function (Beckwitt et al., 2018), and more recently, a simulation of a body-on-a-chip multi-organ system (McAleer et al., 2019) to assess drug efficiency and toxicity.

## Part III—Organotypic Models of Cancer Progression and Drug Response

Understanding the key aspects of tumoral progression is of utmost importance for the development of novel successful anticancer strategies. Organotypic modeling of these aspects alongside the interactions between the different actors of the TME would allow a better comprehension of the mechanisms that mediate tumoral progression and a first solid step toward preclinical drug screening in physiologically relevant situations. In this section, we describe how the previously mentioned organotypic models have been applied to study the different steps of tumor growth and metastasis (Table 1).

**TABLE 1:** Organotypic models used to study cancer progression stages and drug response.

	**Primary tumor growth**	**TME-tumor cells interactions**	**Invasion and migration**	**Angiogenesis and intravasation**	**Extravasation and secondary organ colonization**	**Drug response**
**Multicellular spheroids**	**Tumor growth** Ovarian cancer (Yin et al., 2016) Bladder cancer (Namekawa et al., 2020)	**CAF-mediated interactions** Lung cancer (Chen et al., 2018) Pancreatic cancer (Ware et al., 2016)	**Invasion** Breast cancer (Avgustinova et al., 2016) Colon cancer (Nam et al., 2018) Colorectal cancer (Libanje et al., 2019)	**Vessel sprouting and intravasation** Colon cancer (Ehsan et al., 2014)	**Niche activation and colonization** Breast cancer (del Pozo Martin et al., 2015)	**High-throughput toxicology assay** Breast cancer (Lee et al., 2008)

**Organoids**	**Introduction of carcinogenesis driver mutations** Colorectal cancer (Matano et al., 2015; Takeda et al., 2019) Breast cancer (Dekkers et al., 2020)	**Stromal interactions** Pancreatic cancer (Öhlund et al., 2017) Intestinal cancer (Roulis et al., 2020) **Immune cells-mediated interactions** Colorectal cancer (Dijkstra et al., 2018) Different tumor types and stages (Neal et al., 2018)	**EMT** Breast cancer (Jung et al., 2019) **Invasion and migration** Breast cancer (Zhang et al., 2019; Georgess et al., 2020)	**Angiogenesis** Breast cancer (Wörsdörfer et al., 2019)	**Extravasation** Breast cancer (Fernández-Periáñez et al., 2013) B Cell Lymphoma (Jia et al., 2020)	**Tumor genetic profiling and response to chemotherapy** Rectal cancer (Ganesh et al., 2019) Pancreatic cancer (Tiriac et al., 2018) Colorectal cancer (van de Wetering et al., 2015; Fujii et al., 2016; Ooft et al., 2019) Gastrointestinal cancers (Vlachogiannis et al., 2018) Renal cancer (Calandrini et al., 2020)

**3D-tissues**	**Neoplastic transformation** Multiple epithelia (Ridky et al., 2010) Colon cancer (Chen H. J. et al., 2016)	**ECM influence** Glioblastoma (Sood et al., 2019)	**Invasion** Multiple epithelia (Ridky et al., 2010) Glioblastoma (Koh et al., 2018)	**Angiogenic response** Breast cancer (Mazio et al., 2018)	**Colonization** Breast cancer (Xiong et al., 2015)	**High-throughput drug screening** Hepatocarcinoma (Chen et al., 2010) Breast cancer (Brancato et al., 2018)

**Microfluidic approaches**	**Tumor growth** Breast cancer (Nashimoto et al., 2020)	**CAF-mediated interactions** Breast cancer (Pelon et al., 2020; Truong et al., 2019) Melanoma (Jenkins et al., 2018)	**Invasion and migration** Breast cancer (Chen et al., 2018; Truong et al., 2019) **Migration** Lung cancer (Hsu et al., 2011) Breast cancer (Li et al., 2017)	**Angiogenesis** Microvessels formation and endothelial functions (Zheng et al., 2012) **Angiogenic growth and intravasation** Breast cancer (Zervantonakis et al., 2012; Tang et al., 2017; Sano et al., 2018; Shirure et al., 2018)	**Extravasation** Breast cancer (Jeon et al., 2015; Chen M. B. et al., 2016, 2017) **Metastasis** Breast cancer (Bersini et al., 2014)	**Response to chemotherapy** Lung cancer (Hassell et al., 2017) Breast cancer (Choi et al., 2015)

### Cancer Modeling Using Organotypic Models

#### Tumor Growth *in situ*—Interactions of Cancer Cells With the TME Elements

Many *in vitro* organotypic models have been used to study tumor initiation and growth and to identify how parenchymal cells (endothelial, epithelial, immune, nerve and stromal cells) and components (ECM, secreted factors) of the TME influence the growth *in situ* of different cancer types.

Modeling cancer initiation using organoid is highly attractive owing to the relative ease of genetic manipulation of cells. Using CRISPR-Cas9 genome editing, tumor suppressors have been identified (Michels et al., 2020), as well as the consequences of mutations in the DNA repair deficiency genes (Drost et al., 2017) or mutations that drive cancer progression (Fumagalli et al., 2017) have been elucidated. Such approaches allow the introduction of defined mutations to transform normal organoids and induce tumor growth, upon xenotransplantation. Matano and colleagues model human colon adenocarcinoma by introducing canonical colorectal cancer (CRC) driver mutations into primary human colon organoid cultures (Matano et al., 2015), revealing that mutations in *APC*, *SMAD4*, *TP53*, and *KRAS* simultaneously are sufficient to model colonic adenomas but not tumorigenesis, perhaps due to the lack of TME components within the organoids. Similarly engineered CRC organoids with *APC* and *KRAS* mutations formed dysplasia and could invade submucosa (Takeda et al., 2019), and transformed mammary organoids formed tumors upon xenotransplantation (Dekkers et al., 2020). Thus, deconstructing carcinogenesis into single genetic elements by engineering cancer genes in untransformed human organoids is a powerful tool for investigating how individual genetic aberrations contribute to the acquisition of cancer phenotypes.

Nevertheless, the genetic alterations driving cancer initiation are supplemented by the interactions of cancer cells with their microenvironment to ensure successful cancer progression. A refined cancer 3D-tissue model using cancer-associated genetic modifications and a stromal department showed the neoplastic transformation of normal epithelia which became invasive (Ridky et al., 2010). Indeed, many tumors are characterized by a prominent stromal compartment that modulates tissue architecture, due to extensive ECM remodeling mainly mediated by CAFs. Adding stromal fibroblasts to prostate organoids facilitated their branching (Richards et al., 2019), while the addition of CAFs to lung squamous carcinoma spheroids recapitulated the pathological changes of tumorigenesis, from invasion and hyperplasia to dysplasia (Chen et al., 2018). Additionally, CAFs were shown to enhance invasion and migration of breast cancer cells in a 3D microfluidic device (Nguyen et al., 2018; Truong et al., 2019).

Furthermore, the coculture of pancreatic stellate cells, a resident mesenchymal cell population that differentiates into CAFs, with pancreatic cancer patient-derived organoids (PDOs) (Öhlund et al., 2017) or with spheroids (Ware et al., 2016) produced a highly desmoplastic stroma, typical of pancreatic carcinomas. Equally investigating the role of the TME in CRC initiation using organoids, Roulis and colleagues performed single-cell RNA sequencing of the murine intestinal mesenchymal niche and found a population of fibroblasts in intestinal crypts that orchestrate intestinal tumorigenesis by exerting paracrine control over tumor initiating stem cells (Roulis et al., 2020).

Other key elements of the TME which significantly affect cancer cell behavior are immune cells. Tumor-immune system interactions have been widely studied by culturing immune cells recovered from patients together with established cancer cell lines in conventional monolayer cultures. However, these approaches fail to account for critical aspects of the TME. Indeed, microfluidic devices customized with human tumor spheroids containing immune cells recapitulate some features of response or resistance to immune checkpoint blockade in melanoma (Jenkins et al., 2018), but without features of the stromal compartment. The recent promise of therapies manipulating tumor-infiltrating immune cells created a particular exigency for human cancer models that recapitulate this TME diversity. In an effort to integrate an immune competent microenvironment to organoid cultures, a platform to induce and analyze tumor-specific T-cell responses to epithelial cancers was established (Dijkstra et al., 2018). Enrichment of functional tumor-reactive T lymphocytes from CRC or non-small cell lung cancer (NSCLC) patients was successfully established by cocultures of peripheral blood lymphocytes with autologous tumor organoids. These tumor-reactive T cells efficiently recognize and kill autologous tumor organoids, while leaving healthy organoids unharmed. Moreover, a recent study presents organoid modeling that preserves primary tumor epithelium with its endogenous immune and non-immune stromal elements (Neal et al., 2018).

#### Cancer Progression: EMT, Cancer Cell Migration and Invasion

The metastatic cascade initiates with invasion and migration of tumor cells away from the primary tumor. Invasion through the basement membrane is considered a differentiating step between neoplasia and malignant tumors. Because cancer cell contractility and matrix stiffness are critical parameters for invasion, accurate invasion models should include tunable matrix parameters (Wisdom et al., 2018). This is possible using organotypic 3D tissues, where virtually any component can be readily modulated. The stromal compartment can be enriched not only with fibroblasts but with myofibroblasts, endothelial cells or inflammatory cells (reviewed in Coleman, 2014).

To study the basis of cancer invasion, significant efforts have been made to recapitulate tumor–stroma interactions. Multicellular spheroids combined with ECM containing fibroblasts showed enhanced invasion (Avgustinova et al., 2016). However, the tumor and its environment being highly dynamic, microfluidic approaches are more fitted to study tumor cell migration. Indeed, the use of such approaches unveiled the contributions of different cell types to tumor cell migration and invasiveness. A 3D microfluidic coculture system containing side-by-side tumor and stroma regions showed that CAFs enhanced the migration and invasiveness of cancer cells (Truong et al., 2019; Pelon et al., 2020). TGFβ secreted by cancer cells was shown to stimulate fibroblasts to transform into myofibroblasts, which then produced soluble factors that fed back to increase the migration speed of the cancer cells (Hsu et al., 2011). Likewise, the cytokines secreted by macrophages cocultured with cancer cells in a microfluidic device, increased cancer cell migration speed and persistence in a MMP-dependent fashion (Li et al., 2017).

#### Angiogenesis and Cancer Cell Intravasation

Over the last decade, biomimetic 3D vascular models have been developed, contributing to the understanding of angiogenic processes. Rings of tissue from human umbilical arteries embedded into a 3D matrix were able to sprout in response to tumor-derived proangiogenic factors (Seano et al., 2013). However, vascular organotypic models should not be static as shear forces and blood flow are important for the vascularization process. So, microfluidic approaches have been developed in which endothelial cells are seeded into a channel within ECM to form a primitive vasculature that can be stimulated by angiogenic factors (Zheng et al., 2012; Nguyen et al., 2018), or with an incorporated layer of human bone marrow stromal cells around the channels to recapitulate perivascular barrier function (Alimperti et al., 2017). These microfluidic chips can also be used to trigger vasculogenesis; in that case, instead of seeding endothelial cells beside the matrix, endothelial cells, fibroblasts (Jeon et al., 2014) and tumor cells (Chen M. B. et al., 2017) are loaded within the matrix. Moreover, the ability of organoid-on-a-chip to mimic perfusable blood vessels may address an important issue of organoid use: the lack of nutrient supply. To surmount this, a tumoroid-on-a-chip was developed. It was created in a microfluidic device consisting of three interconnected chambers that enable the self-assembly of endothelial cells into a 3D network of blood vessels and their angiogenic growth toward the organoid-like structures from breast cancer patients (Shirure et al., 2018). However, in such approaches, endothelial cells may not always be free to interact with tumor cells because of the artificial membranes used in the organ-on-a-chip devices. To address this issue, endothelial cells were modified to produce ‘reset’ vascular endothelial cells (R-VECs) that grew into 3D branching vessels capable of transporting human blood in microfluidic chambers and when transplanted into mice (Palikuqi et al., 2020). These R-VECs adapted their growth upon their coculture with either normal colon organoids or patient-derived colorectal organoids. They arborized normal colon organoids and helped sustain their proliferation while they erratically infiltrated tumor-derived organoids, thus providing a novel physiological platform to study vasculogenesis and angiogenesis.

Entry of tumor cells into the blood stream is a critical step in cancer metastasis. Using microfluidic devices, interactions between invasive cancer cells and endothelial cells have been studied. It was shown that treatment of the endothelium with TNF or coculture with macrophages resulted in rapid and increased numbers of tumor cell–endothelial cell attachment events (Zervantonakis et al., 2012). The secretion of cytokines and chemokines by cancer cells increases the permeability of the endothelial barrier, allowing tumor cells to intravasate and extravasate (Reymond et al., 2013). This feature was modeled using a perfused microfluidic platform containing a vascular compartment with breast cancer cells and their associated endothelial cells separated via a micropillar array interface that allows direct communication of tumor and endothelial cells. The permeability of the vessels was greatly increased in response to the presence of tumor cells or tumor cell-conditioned medium (Tang et al., 2017). Moreover, a tissue-engineered model containing a realistic microvessel in coculture with mammary tumor organoids allowed real-time monitoring of tumor cell-vessel interactions. Using this model, it was shown that tumor cells can reshape, destroy, or intravasate into blood vessels (Silvestri et al., 2020).

#### Extravasation and Secondary Site Colonization

Cancer cells within vessels must extravasate to colonize new sites. This process is different from intravasation, because the vasculature to be breached is healthier and cancer cells experience fluid shear stresses due to blood flow. After extravasation, cancer cells have one final task to complete: colonization of secondary sites. Extravasation of tumor cells has been shown to occur *via* endothelial apoptosis *in vitro* (Heyder et al., 2002) but *via* necroptosis *in vivo* (Strilic et al., 2016). Thus, accurate modeling of the extravasation and colonization steps requires tissue-specific cell types, microenvironmental cues, and vascularization. Breast cancer cells extravasated through a vascular network into a bone-mimicking microenvironment generated by culturing osteo-differentiated MSCs within a hydrogel, or within a microfluidic device (Jeon et al., 2015; Sano et al., 2018). It was shown that extravasation rates were much higher to the bone microenvironment than to stromal matrices alone. Another similar model showed that β1 integrin expression is required for cancer cells to be able to invade through the endothelial basement membrane (Chen M. B. et al., 2016). Increased complexity and clinical relevance can be incorporated into organ-on-a-chip models, as devices have been developed to mimic interactions between circulating tumor cells (CTCs), endothelium and bone microenvironments as a model of metastasis to bone (Bersini et al., 2014).

### Therapeutic Applications of Organotypic Models

Although the demand for anticancer drugs is constantly increasing, their development is slow and fastidious. Monolayer cultured cells are the most widely used *in vitro* models despite their inability to accurately reflect drug’s metabolism and pharmacokinetics in the human body. For years, cell-based drug discovery was based on monolayer cultures of authenticated cell lines (Smith et al., 2010; Barretina et al., 2012), but in this blooming era of precision medicine (Prasad et al., 2016), organotypic models represent great promise for anticancer drug discovery.

In line with this, using an organ-on-a-chip approach, a human lung cancer chip has been developed to study tumor growth patterns and drug response (Hassell et al., 2017). When lung cancer cells were cultured within a physiological-like microenvironment composed of lung endothelial cells, normal lung alveolar epithelium and ECM, they presented rampant growth and resistance to tyrosine kinase inhibitors (TKI) similar to NSCLC patient’s response, while they failed to do so in static conventional culture. Likewise, McAleer and colleagues designed a modulable five-chamber multi-organ system to monitor drug effects and simultaneously examine anticancer drug efficacy and off-target toxicity (McAleer et al., 2019). In two models incorporating an array of cancer and healthy human cell types, the system provided insight into the efficacy and toxicity of diclofenac, imatinib, and tamoxifen.

Beyond engineered organoids, organoids derived from patient biopsies or resected tumors, called patient-derived organoids (PDOs) have been successfully cultured with a high success rate and indefinite expansion. These contain tumor cells and stromal cells, thus providing a more realistic microenvironment and they seem to retain the tissue identity of the patient (Tiriac et al., 2018; Ganesh et al., 2019), indicating their great potential for personalized medicine approaches. Recent studies suggest that PDOs mirror clinical responses of individual patients to therapy within a clinically meaningful timeframe and even predict patient response to chemotherapy (Ooft et al., 2019; Pasch et al., 2019). Indeed, PDOs derived from glioblastoma samples were used to test responses to standard of care therapy as well as targeted treatments, like chimeric antigen receptor T (CAR-T) cell immunotherapy in a clinically relevant timescale (Jacob et al., 2020). These PDO properties laid the foundation of what is now known as organoid biobanks (van de Wetering et al., 2015; Calandrini et al., 2020) used for applications such as drug testing, cytological analyses, and xenografting.

With the significant need for biomarker identification of drug response, PDOs could also be considered as a tool for biomarker discovery by analyzing secreted factors such as extracellular vesicles (Huang L. et al., 2020) in contrast with PDX models, due to the presence of contaminating host factors. Although molecular diagnostic testing is now routinely used to determine the choice of targeted therapies for the treatment of cancer patients, patients in advanced stages who have exhausted standard clinical care approaches lack personalized therapeutics and will endure the arduous regimen of chemotherapy and see little or no benefit. Even if the use of functional testing in guiding personalized medicine in still in its infancy, the use of metastatic cancer site derived PDOs to evaluate drug response has proven its efficacy by recapitulating patient response (Weeber et al., 2015; Fujii et al., 2016; Pauli et al., 2017; Vlachogiannis et al., 2018). These evaluation platforms could be of great interest in orienting the treatment of advanced cancer patients.

### Shortcomings and Future Directives of Organotypic Models in Translational and Preclinical Settings

The use of organotypic models for cancer modeling is a blooming area of research, however, there are still limitations to their use (Puca et al., 2018; Fujii and Sato, 2020). As an example, studying angiogenesis is rudimentary when it comes to organotypic models. Indeed, the use of vasculature is very basic and organotypic models with other surrounding tissue types are necessary to model more physiological situations. It would be of great interest to model angiogenesis and neovascularization within a transformed organoid. Additionally, complexifying organotypic models by engineering organoids surrounded by muscle, an immune system, and containing a neuronal network along with functional vasculature is something to look forward to in the near future.

When it comes to preclinical studies, organotypic models face many caveats. Spheroid-based 3D models must be used with caution when it comes to clinical relevance. Because they are generated from non-primary tumor cell lines (Friedrich et al., 2009), their use should be restricted to signaling pathways, mechanistic studies and first-line HTS drug screens. More sophisticated models like organoids could be used to validate drug candidates. Stem cell-derived organoids are important for modeling epithelial tumors. However, the lack of standardization and quality control of stem cell culture are an obstacle for their use in clinical studies. The use of pluripotent stem cells for organoid generation can be hampered by the presence of contaminating progenitors that can yield undesired cell types and a small population of undifferentiated PSCs can give rise to tumors that out-compete organ reconstitution *in vivo* (Fowler et al., 2020). Furthermore, due to different culture methods, organoids may present undesired phenotypic variabilities. Interestingly, the recent development of microwell arrays in a matrix-free solid manner allowed the high-throughput assessment of homogenous organoids in 3D culture (Brandenberg et al., 2020). The most exciting aspect for organoid use in clinics is the implementation of PDOs for personalized medicine but this requires that pure PDO cultures can be established, which is not always the case. For example, prostate cancer organoids can only be generated from metastases because normal prostate epithelial cells overgrow cancer cells (Gao et al., 2014). Additionally, the majority of organoids derived from intrapulmonary tumors were overgrown by normal airway organoids (Dijkstra et al., 2020), hampering their use for preclinical studies. Nonetheless, evidence of divergence from primary tumors emerged over time with a decreased abundance of populations from the TME coupled with lower expression of immune-related genes in PDOs (Jacob et al., 2020). Future studies are needed to improve this issue and to maintain the immune compartment, notably for relevant testing of immunotherapies in PDO biobanks.

Microfluidics require refined technical innovations to enable scaling up for HTS. Integration of organotypic models, spheroids, organoids or PDOs, with simulated physiology in microfluidic platforms could represent one of the most relevant *in vitro* models. Two very exciting studies recently reported a near complete body-on-a-chip system. One described an eight organ-chip model linked via vascular endothelial-lined compartments: gut, liver, heart, kidney, lung, heart, brain, blood-brain barrier, and skin (Novak et al., 2020). Using the same approach, intravenously administered cisplatin *via* an arteriovenous reservoir, provided clinically relevant results when compared to *in vivo* behavior (Herland et al., 2020). In this regard, the microfluidic field is still maturing, with a need for regulatory guidelines among the scientific community, specifically for the validation of organ-on-a-chip technology for pharmacological drug testing.

## Concluding Remarks

Understanding tumors, now considered as heterogenous abnormal organs, is insufficient if the tumor cells are studied individually. Methods that are more inclusive are needed that integrate the cellular, genomic, microenvironmental and spatial features of cancers to be able to understand and overcome their numerous resistance mechanisms. Increasing the complexity of the used models lead to the development of many organotypic cancer models that are physiologically relevant and allow in-depth understanding of the interactions that take place within a tumor. Moreover, future studies are needed to standardize organoid culture methods across the scientific community, as this is very heterogenous at the moment. It is also needed to enhance such cultures by adding stromal and immune compartments to organoid culture to better mimic the tumor microenvironment. This is important because patient-derived organoids represent a very promising approach for personalized medicine, as they retain patient and tumor identity and mirror drug response, thus allowing the use of tailored medicine and avoiding the use of unnecessary treatments. Such organoids, cryo-preserved and collected to form biobanks, should they be available to the scientific community, may replace conventional drug screening assays because they fit the requirements of automated high-throughput screenings. More sophisticated organotypic models, fruits of the collaboration between biologists and engineers, could represent the future of cancer research. Multi-organoid systems also referred to as “body-on-a-chip” will enable the development of biologically complex systems, where organoids derived from different tissues are brought together and allowed to integrate, mimicking organ function and allowing disease modeling.

## Author Contributions

OM conceived the review outline. MH wrote the manuscript and made the figures with support from OM. CN and CV contributed to the final manuscript. All authors contributed to the article and approved the submitted version.

## Conflict of Interest

The authors declare that the research was conducted in the absence of any commercial or financial relationships that could be construed as a potential conflict of interest.

## Footnotes

1 https://www.nc3rs.org.uk/the-3rs
